# Assessment of Orthographic Similarity of Drugs Names between Iran and Overseas Using the Solar Model

**Published:** 2017-12

**Authors:** Nazanin ABOLHASSANI, Ali AKBARI SARI, Arash RASHIDIAN, Mansoor RASTEGARPANAH

**Affiliations:** 1. Dept. of Health Management and Economics, School of Public Health, Tehran University of Medical Sciences, Tehran, Iran; 2. Dept. of Clinical Pharmacy, College of Pharmacy, Tehran University of Medical Sciences, Tehran, Iran

**Keywords:** Patient safety, Drug proprietary names, Orthographic similarity, General office of trademarks registry, Solar model, Iran

## Abstract

**Background:**

The recognition of patient safety is now occupying a prominent place on the health policy agenda since medical errors can result in adverse events. The existence of confusing drug names is one of the most common causes of medication errors. In Iran, the General Office of Trademarks Registry (GOTR), for four years (2010–2014) was responsible for approving drug proprietary names. This study aimed to investigate the performance of the GOTR in terms of drug names orthographic similarity using the SOLAR model.

**Methods:**

First, 100 names were randomly selected from the GOTR’s database. Then, each name was searched through pharmaceutical websites including Martindale (the Complete Drug Reference published by Pharmaceutical Press), Drugs.com and Medicines Complete. Pair of drugs whose names look orthographically similar with different indications were identified. Then, the SOLAR model was utilized to determine orthographic similarity between all pair of drug names.

**Results:**

The mean of match values of these 100 pairs of drug was 77% indicating the high risk of similarity. The match value for most of the reviewed pairs (92%) was high (≥66%). This value was medium (≥ 33% and <66%) just for 8% of the pairs of drug. These results indicate high risk of confusion due to similarity of drug names.

**Conclusion:**

The stewardship of the GOTR in patient safety considerations is fundamentally problematic. Thus, as a best practice, we recommend that proprietary names of drugs be evaluated by an entity within the health system. While an entity within the health system should address patient safety considerations, the GOTR is responsible for intellectual property rights.

## Introduction

The recognition of the importance of patient safety is now increasing and garnering renewed regulatory interest since medical errors can result in adverse events from the inappropriate therapy (1). As clinical medicine is a hugely complex field, the occurrence of errors is unsurprising. Whilst is not a new phenomenon, medication errors may occur in all healthcare systems and are a common threat to patient safety (2). In this regard, the existence of confusing medication names is regarded to be one of the most common reasons for medication errors and is of concern throughout the world (3). Attention to the issue of drug name confusion has also been mentioned within a set of nine Patient Safety Solutions (4). Medication errors due to orthographic similarity of drug names underscore the serious nature of this type of error that indicates the need for considerable attention and regulation to restrict such errors. This issue is also introduced as one of research priorities in Iran (5).

WHO has a constitutional mandate to “develop, establish and promote international standards with respect to biological, pharmaceutical and similar products”. This Organization collaborates with International Nonproprietary Name (INN) experts as well as national nomenclature committees in order to select a single name for each medication that should be of worldwide acceptability. While generic medicines are marketed under a non-proprietary or approved name instead of a proprietary or brand name, the Trade-Related Aspects of Intellectual Property Rights (TRIPS) agreement does not hinder countries from allowing generic substitution. Besides, competition between pharmaceutical companies and generic producers has been regarded to be more effective than negotiations with pharmaceutical companies in reducing the cost of drugs (6).

Full responsibility of the process of screening and approving drug proprietary names was delegated to the General Office of Trademarks Registry (GOTR) within the State Organization for Registration of Properties for a four-year time interval (2010–2014) and this entity was responsible for approving drug proprietary names before market entry. The naming principle consists of two components: ‘patient safety issue and intellectual property rights’.

This study aimed to investigate the performance of the GOTR in terms of drug names orthographic similarity using the SOLAR model.

## Materials and Methods

To calculate orthographic similarity of drug proprietary names approved by the General Office of Trademarks Registry (GOTR), first 100 drug proprietary names were randomly selected from the GOTR’s database. Then, each name was searched through pharmaceutical websites including Martindale (the Complete Drug Reference published by Pharmaceutical Press), Drugs.com and Medicines Complete. Through this search, pair of drugs whose names look orthographically similar were identified. In addition, indications for Iranian approved drug names and those drug names exist in the world were reviewed. In the case of similar indication, the identified similar pair of drug was omitted and was replaced by another similar pair of drug with different indication. Then, the self-organizing lexical acquisition and recognition (Solar) model of visual word recognition was utilized to determine orthographic similarity between all pair of drug names. The Solar model was used because of its capacity for stable self-organization, its spatial coding scheme, its combination of serial and parallel processes, and its chunking mechanism. The model also introduces a novel mechanism to explain word frequency effects. Another distinctive feature of the model is its incorporation of a novel opponent processing mechanism for performing lexical decision (7). The orthographic similarity values for the 100-drug name pairs based on the SOLAR models were ranked using the following formula:

A spatial code can be written as a vector consisting of n elements, where n is the number of letters in the input string and the values in the vector represent the activities of the corresponding letter nodes. Spatial codes always use a monotonically descending series to code letter position. For the i^th^ word node, this set of letters is denoted L_i_, and the number of letters in this set (i.e., the length of the word) is denoted l_i_. The weight between a letter node and a word node is equivalent to the value of that letter node’s activity in the spatial code for that word.

Therefore, the first step involves computing a set of signal-weight differences. For each of the elements in the set L_i_ a difference d_ji_ is computed by subtracting from s_j_ (the activity of the j^th^ letter node) the corresponding weight z_ji_, i.e.,

(1) $${\text{d}}_{\text{ji}}={\text{s}}_{\text{j}}-{\text{z}}_{\text{ji}}$$

Each signal-weight difference is then associated with a continuous function f_ji_(x) that is symmetrical around x = d_ji_:

(2) $${\text{f}}_{\text{ji}}(\text{x})={\text{e}}^{\left(-(\text{Dji}-\text{x})2/\sigma \right)}$$

The parameter σ in (2) controls the width of the difference function and can be interpreted as a measure of letter position uncertainty (a default value of σ = 3 is assumed for this parameter). Then the superposition of these functions is:

(3) $${\text{F}}_{\text{i}}(\text{X})={\sum_{\text{j}}\in }_{\text{Li}}{\text{f}}_{\text{ji}}(\text{x})$$

(Where the set Li refers to the set of comparison letters). A match value M_i_ can then be found by dividing the peak of the superposition function by the number of comparison Letters (l_i_), that is,

(4) $${\text{M}}_{\text{i}}\frac{\text{max}(\mathrm{Fi}(x))}{l\text{i}}$$

The set of equations 1 through 4 produce a match value that lies between 0 and 1. This value is stated as a percentage and three threshold values, low (≥ 1%), medium (≥33%), and high (≥66%) were used to determine whether the pairs of drug names were connected based on their orthographic similarity ratings (8).

## Results

The results of Table 1 show the match values between each drug proprietary name approved by the GOTR and the drug name looking similar to that. The match value ≥ 66 is considered as high risk of similarity based on the Solar ranking. The mean of match values of these 100 pairs of drug was 77 indicating the high risk of similarity.

**Table 1:** Match values and rate of risk associated with pair of drugs

***No.***	***Nonproprietary name (Generic name)***	***Proprietary name (in Iran)***	***Similar name in the world with different indication/Generic name written in parenthesis***	***Match value (%)***	***Rate of risk confusion***
1	ACA	AXAR®	RAXAR (grepafloxacin)		High
2	ACETAMINOPHEN	TINYPHEN®	SINIPHEN (Caffeine, Propyphenazone, Salicylamide)	70	High
3	ACETAMINOPHEN/CAFFEINE/IBUPROFEN	RAHAFEN®	RAPIFEN (Alfentanil)	78	High
4	ADULT COLD PREPARATION-5	FARALEX®	FARMALEX (Cefalexin)	91	High
5	ADULT COLD PREPARATION-7	ZOCAMAX®	TOPAMAX (topiramate)	67	High
6	ADULT COLD PREPARATION-4	EXACOLD®	DEXACOL (Dexamethasone)	67	High
7	ADULT COLD	GRIPHEN®	PRIPHEN (Nandrolone)	78	High
8	AMANTADINE HCL	AMMOREL®	AMIOREL (Bromhexine)	89	High
9	AMLODIPINE/ATORVASTATIN	TENSOLIP®	TENSOLIV (chlordiazepoxide-clidinium)	80	High
10	ARIPIPRAZOLE	SEROZOL®	SEROZIL (Cefprozil)	89	High
11	ATORVASTATIN	ATOSTROL®	HALOSTROL (halobetasol propionate)	80	High
12	AZATHIOPRINE	AZARAM®	AZACTAM (aztreonam)	81	High
13	BECLOMETHASONE DIPROPIONATE	BECLORHIN®	BECLOTRIN (Betametasona +Clotrimazol+ Gentamicina)	88	High
14	BUSERELIN ACETATE	CINNAFACT®	CINNAPAC (Cinnarizine)	73	High
15	CALAMINE	CALAMEX®	CALMEX (Doxylamine)	80	High
16	CALCIUM FOLINATE	ROFOLIN®	ROFOXIN (Ceftriaxone: (ceftriaxone sodium and dextrose)	89	High
17	CEFIXIME	LOPRAX®	LOPROX (Ciclopirox)	88	High
18	CEFTIZOXIME SODIUM	AFAZOX®	AFAZOL (naphazoline hydrochloride)	75	High
19	CETIRIZINE 2HCl	CETRIKIM®	CETROTIDE (Cetrorelix)	60	Medium
20	CETIRIZINE/PSEUDOEPHEDRINE	CETADIN®	CEFADIN (Cephalexin)	89	High
21	CIPROFLOXACIN HCL	CIPLEX®	IPLEX (mecasermin rinfabate)	75	High
22	CITALOPRAM HBR	BIOXAL®	BIOXTRA (saliva substitutes topical)	66	High
23	CLOBUTINOL HCL	TIDOCAUGH®	ETIDOCAINE (Etidocaine)	55	Medium
24	CLOPIDOGREL	DIPIX®	DEPIXOL (Flupentixol)	71	High
25	CO TRIMOXAZOLE	DUCOTRI®	DUCORT (Deflazacort)	72	High
26	COLCHICINE	MODACINE®	MODACIN (Ceftazidime pentahydrate)	80	High
27	CONTRACEPTIVE HD	OVUSTOP-H®	ACUSTOP (Flurbiprofen)	45	Medium
28	DEFERASIROX	OSVERAL®	FEVERALL (Acetaminophen)	67	High
29	DIAZEPAM	ZEPADIC®	ZEPATIER (elbasvir and grazoprevir)	67	High
30	DICLOFENAC SODIUM	DICLEN®	DICLEGIS (doxylamine and pyridoxine)	75	High
31	DIMETHICONE	DILICE®	DILOMINE (dicyclomine)	69	High
32	DOMPERIDONE MALEATE	MOTIDON®	METADON (methadone)	78	High
33	ESOMEPRAZOLE	MAXOPRAZOL®	MEDOPRAZOLE (Omeprazole)	75	High
34	EXPECTORANT	COUFEX®	KEFLEX (Cephalexin)	47	Medium
35	EZETIMIBE	EZITAL®	EMITAL (Ondansetron)	88	High
36	FEXOFENADINE	ALLEXAFEN®	ALEXAN (Cytarabine)	61	Medium
37	FURAZOLIDONE	FURABEN®	FURACIN (nitrofurazone)	78	High
38	GALANTAMINE	ALZAMIN®	ALAMIN (PHENYLEPHRINE HCL/CHLORPHENIRAMINE MALEATE)	83	High
39	GEMCITABINE (as HCL)	CHEMOGEM®	CHEMOFER (Folic Acid, Iron, Vitamin B12)	70	High
40	GLATIRAMER ACETATE	OSVIMER®	OSIMERTINIB (osimertinib)	78	High
41	GRANISETRON	GRATRIL®	GABITRIL (tiagabine HCl)	78	High
42	HEPARIN SODIUM	CLOTIN®	CLOPINE (clozapine)	75	High
43	HYDROXYPROGESTERONE CAPROATE	FEMOLIFE®	MEMOLIFE (Vitamins and minerals)	80	High
44	IBUPROFEN	ACTOPIN®	ACTAPIN (Amlodipine)	89	High
45	IMIPENEM+CILASTATIN	CILAVIL®	CILARIL (Cilazapril)	89	High
46	INTERFERON ALFA-2B	PDFERON®	MYFERON (polysaccharide-iron)	67	High
47	IOHEXOL	OPAQUESOL®	ELDOPAQUE (Hidroquinone)	55	Medium
48	ISOXSUPRINE HCL	ISUPRINE®	ISUDRINE (aluminum phosphate & magnesium oxide)	90	High
49	LATANOPROST+TIMOLOL	COPROST®	CARBOPROST (carboprost)	90	High
50	LETROZOLE	LETRAX®	LETROX (levothyroxine)	88	High
51	LEVOFLOXACIN	TAVANEX®	TAVINEX (Ambroxol)	89	High
52	LIDOCAINE	XYLEX®	XYLAREX (d-xylitol)	79	High
53	LISINOPRIL	LISIREX®	LIPIREX (Atorvastatin)	89	High
54	LOSARTAN POTASSIUM	CARDIOSAN®	CARDIOGEN (cardiogen)	82	High
55	LOSARTAN/HYDROCHLOROTHIAZIDE	HYDROZAAR®	HYDROPANE (homatropine/hydrocodone)	64	Medium
56	MEBEVERINE	COLIBSAN®	COLISTIN (colistimethate sodium)	77	High
57	MEDROXYPROGESTERONE ACETATE	DEPOGESTRONE®	DEPO-TESTOSTERONE (testosterone cypionate)	70	High
58	MEDROXYPROGESTERONE ACETATE	PROVEDIC®	PROVENTIL (albuterol sulfate)	67	High
59	MELOXICAM	ROMATOBIC®	CROMATONBIC (Calcium Folinate, Vitamin B12)	83	High
60	METFORMIN HCL	METOVER®	MEROVER (meropenem)	89	High
61	METHOCARBAMOL	RELAXIMOL®	RELAXOL (Citalopram)	69	High
62	METOCLOPRAMIDE	PLADIC®	PLACIDYL (Ethchlorvynol)	69	High
63	METOPROLOL TARTRATE	TEDAPROL®	TEDAROL (Triamcinolone Diacetate)	82	High
64	MUPIROCIN	AFIROCIN®	AZITROCIN (azithromycin)	85	High
65	NANDROLONE DECANOATE	DECANDROLONE®	OXANDROLONE (Oxandrolone)	71	High
66	NAPHAZOLINE ANTAZOLINE	ANAPRIVIN®	ANAPRILIN (propranolol)	91	High
67	NAPROXEN	NAPTIN®	NAFTIN (naftifine hydrochloride)	88	High
68	NITROGLYCERIN SR	TRICONTIN®	TRICON (Fluconazole)	73	High
69	OLANZAPINE	ZYPROBIOX®	PROBIOX (Ciprofloxacin)	73	High
70	ORLISTAT	XENOLIP®	FENOLIP (fenofibrate)	78	High
71	OXYTOCIN	OXYTIP®	OXYVIT (Retinol)	66	High
72	PANTOPRAZOLE	PENTOMID®	PENTOXIL (pentoxifylline)	70	High
73	PANTOPRAZOLE	PANTOSS®	PANTOSSE (Ethylmorphine)	89	High
74	PENTOXIFYLLINE	PENTAFIL®	PENTACEL (pentacel)	80	High
75	PHENYLEPHRINE	NEOPHRIN®	NEOPYRIN (Acetaminophen)	90	High
76	POLYETHYLENGLYCOL	COLOLAX®	CODOLAX (chlorpheniramine maleate/codeine phosphate/papaverine hydrochloride)	89	High
77	PRASUGREL	BIOSUGREL®	BIOSURE (Amikacin)	68	High
78	PROPYLTHIOURACIL	PROURACIL®	FLUOROURACIL (Fluorouracil)	82	High
79	QUETIAPINE	BIOQUETIN®	BIOQUIN (Hydroquinone)	69	High
80	RALOXIFENE	RALOFEN®	TALOFEN (promazine hydrochloride)	78	High
81	RANITIDINE	ARY-TAC®	ARATAC (amiodarone hydrochloride)	78	High
82	RILUZOLE	RILONORM®	MILONORM (milonorm)	80	High
83	RIVASTIGMINE	CHOLINUP®	CHOLINE (B vitamins)	70	High
84	SOLIFENACIN	SOLIFEX®	SYLIFEX (Silymarin-phospholipides)	89	High
85	SUCCINYLCHOLINE CHLORIDE	MIOKOLIN®	MONOLIN (isosorbide mononitrate)	77	High
86	TADALAFIL	TIAGRIX®	TIARIX (Paroxetine)	80	High
87	TAMSULOSIN	MODALUSIN®	MODAFINIL (Modafinil)	57	Medium
88	TESTOSTRONE ENANTATE	ANDRONE®	ANDROLONE (Nandrolone)	84	High
89	THEOPHYLLINE	THEOMEX®	THEOMAX (ephedrine sulfate/hydroxyzine hydrochloride/theophylline)	89	High
90	TIZANIDINE	SPALEX®	SALEX (Salicylic acid)	84	High
91	TOPIRAMATE	CONVEX®	CONEX (dexbrompheniramine/pseudoephedrine)	81	High
92	TRANEXAMIC ACID	TRANCID®	RANCID (Magnesium Hydroxide)	78	High
93	TRIAMCINOLONE ACETONIDE	CORTIRAN®	CORTIRON (Desoxycortone)	90	High
94	TRIPTORELIN ACETATE	MICRORELIN®	MICROGESTIN (ethinyl estradiol/norethindrone)	79	High
95	VENLAFAXINE	DEPRILAX®	PERILAX (Eperisone)	75	High
96	VITAMIN A	A-VIGEL®	DIVIGEL (estradiol gel/Hormone)	75	High
97	VITAMIN B1/B6/B12	NOROBIT®	NORBIT (Norfloxacin)	80	High
98	VITAMIN B12	VIBALMIN®	VISALMIN (chloramphenicol)	90	High
99	ZOLPIDEM	RAPIDEM®	RAPIDE (diclofenac potassium)	78	High
100	ZONISAMIDE	ZONITED®	ZONITE (Thymol/Propylene Glycol/Benzalkonium/Edetic Acid/Sodium Acetate/Menthol)	78	High
Mean and Standard Deviation				77±0.09	

The first column displays the generic name of the proprietary drugs name (second column) which approved in Iran. The next column shows the similar overseas drug names, with different indication of the local ones, which can be generic or proprietary names (in the case of proprietary names, the generic names have been written in parenthesis). The last two columns show the match values (orthographic similarity rate) and the rate of risk, respectively, based on the Solar (orthographic match value model used in this study). However, some of overseas drug may not exist in Iran pharmaceutical market; however, some similar pairs are generic names that are available in Iran (such as ETIDOCAINE, OSIMERTINIB, CARBOPROST, CARDIOGEN, PENTACEL, FLUOROURACIL, MILONORM, and MODAFINIL). Furthermore, with regard to the existence of many representative pharmaceutical companies which import drugs into Iran, overseas proprietary drugs are also available in Iran market (TOPAMAX, BIOXTRA, CHOLINE, TALOFEN, DIVIGEL, to name a few). Furthermore, the import of any of these overseas drugs is probable.

## Discussion

The current findings indicate the high level (92) of orthographic similarity between local drug proprietary names and overseas drug names. However, there are limited studies on the actual rate of this type of medication error in Iran. The similar names of drugs as the main factor affecting medication errors, so that in 23.40 of the medication errors have been associated with drugs similar names (9). In another study conducted in Iran, the similar drug name was considered as the first factor (36.9) contributing to medication errors in 6 wards of hospital setting (10). In this regard, in a study, in hospitals (including tertiary, university, secondary and primary hospitals) in Thailand, a total of 5327 pairs of medicines were identified as Look-alike Sound-alike medicines (11). Moreover, in Canada, 186 LASA (Look-alike Sound-alike) drug pairs from 3320 possible pairs were identified using the Bigram Similarity algorithm (12). Thus, the issue of confusing drug name is a worldwide issue. In addition, up to 25 of all medication errors were attributed to name confusion (13). Generally, while the similarity in drug names is a worldwide issue, according to the existence of high rate of similar drugs in Iran should be considered by health policy makers.

The performance of the GOTR in terms of screening similar names and patient safety consideration was too weak. One reason for such a weak performance can be the nature of the GOTR; the GOTR, compared to entities within the health system is fundamentally a different entity with various functions. The GOTR’s employees are not individuals with sufficient mastery in the field of medicines and they are not even health professionals. Accordingly, there were many failures in the performance of the GOTR from a patient safety viewpoint. The responsibility for screening drug names in developed countries is always associated with an entity related to health system. Medicines and Healthcare Products Regulatory Agency in UK (14), Food and Drug Administration in USA (15), Health Products and Food Branch in Canada (16) and Therapeutic Goods Administration in Australia (17), to name a few. In this regard, the need for enhanced approval systems for medicine names were emphasized (18). Based upon our discussion so far, the delegation of the responsibility of approving drug names to an entity outside of the health system can result in problems in patient safety.

Limitation of the study: inevitably, our study has not been able to answer a number of questions and in fact has revealed a few new ones. Further research is therefore required. For example, in the future, the question has to be answered is the number of medication errors occurred in Iran due to the orthographic similarity of proprietary drug names. Another issue has to be addressed is the severity of risks due to orthographic similarity of drug names and their impact on patient safety.

## Conclusion

Naming principle consists of two components: ‘patient safety issue and intellectual property rights’. However, the GOTR’s function is necessary for initial naming of drugs and before market entry, it terms of intellectual property rights, it cannot ensure medication safety. Thus, as a best practice, we recommend that proprietary names of drugs be evaluated by an entity within the Food and Drug Organization and the health system. While an entity within the health system should address patient safety considerations, the GOTR can be responsible for intellectual property rights. However, these two elements are different in theory, but in practice not black-white and eventually both should be guaranteed. Furthermore, pharmaceutical manufacturers should meet international criteria (mainly WHO) regarding proposed names, however, there is no guarantee and an entity is still needed to monitor the pharmaceutical manufacturers’ adherence to such criteria.

## Ethical considerations

Ethical issues (Including plagiarism, informed consent, misconduct, data fabrication and/or falsification, double publication and/or submission, redundancy, etc.) have been completely observed by the authors.
